# Safety, tolerability, pharmacokinetics, and pharmacodynamics of LW402, a preferential Janus kinase 1 inhibitor in healthy volunteers: a randomized, double-blinded, placebo-controlled phase 1 trial

**DOI:** 10.3389/fphar.2026.1784350

**Published:** 2026-04-29

**Authors:** Yuzhou Gui, Fan Jiang, Liang Xin, Zhe Wang, Huijuan Zhu, Yanmei Liu, Qian Chen, Jingying Jia, Yun Liu

**Affiliations:** 1 Drug Clinical Trial Center, Shanghai Xuhui Central Hospital/Xuhui Hospital, Fudan University, Shanghai, China; 2 Shanghai Engineering Research Center of Phase I Clinical Research and Quality Consistency Evaluation for Drugs, Shanghai, China; 3 Shanghai Longwood Biopharmaceuticals Co., Ltd., Shanghai, China

**Keywords:** JAK1 inhibitor, LW402, pharmacodynamics, pharmacokinetics, phase I clinical trial, safety

## Abstract

**Introduction:**

This clinical trial aimed to assess the safety, pharmacokinetics (PK), and pharmacodynamics (PD) of LW402, a preferential JAK1 inhibitor, in healthy participants, so as to provide support for its further development in treating autoimmune diseases.

**Methods:**

The study included two phases: a single ascending dose (10–400 mg) with 54 participants and a multiple ascending dose (50–150 mg, b.i.d for 6 days plus a single dose on day 7) with 36 participants. Safety, PK (from timed blood samples), and PD (JAK1/JAK2 signaling) were evaluated.

**Results:**

No serious adverse events were reported; only mild treatment-emergent adverse events (TEAEs), such as sinus bradycardia and gastrointestinal issues, occurred. LW402 was rapidly absorbed (median t_max_: 0.42–1.0 hours), with slightly super-proportional exposure and minimal accumulation, and preferentially inhibited JAK1-mediated signaling.

**Discussion:**

LW402’s favorable safety profile, predictable PK characteristics, and selective JAK1 inhibition collectively support its continued development for the treatment of autoimmune diseases.

**Clinical Trial Registration:**

http://www.chinadrugtrials.org.cn, identifier CTR20201897.

## Introduction

Rheumatoid arthritis (RA) is a chronic progressive autoimmune disease with a global incidence of 0.5%–1% (0.42% in mainland China, ∼5 million patients), characterized by synovitis, pannus formation, and articular cartilage/bone erosion, leading to joint deformity and dysfunction (Smolen et al., 2016). Current therapies (NSAIDs, glucocorticoids, traditional DMARDs, and biological agents) have limitations, including severe adverse effects, poor tolerability, high cost, or parenteral administration (Aletaha and Smolen, 2018; Di Matteo et al., 2023). Thus, the development of safe, effective, and affordable oral targeted small-molecule drugs for RA is an unmet clinical need.

The Janus kinase (JAK)-STAT signaling pathway is essential for transmitting signals from various cytokines and growth factors involved in immune responses, inflammation, hematopoiesis, and cell growth (Shawky et al., 2022; Tanaka et al., 2022). By inhibiting the JAK-STAT pathway, small-molecule drugs block downstream pro-inflammatory and proliferative signaling cascades critical for RA pathogenesis. Specifically, pro-inflammatory cytokines (e.g., IL-6, IL-12, IL-23) bind to their cell surface receptors, triggering the dimerization and activation of JAKs (JAK1, JAK2, JAK3, TYK2) associated with the receptor intracellular domains. Activated JAKs phosphorylate receptor tyrosine residues, creating docking sites for STAT proteins (STAT1, STAT3, STAT5). Phosphorylated STATs dimerize and translocate to the nucleus, where they bind to specific DNA response elements to regulate the transcription of pro-inflammatory genes (e.g., TNF-α, IL-1β, IL-6) and pro-proliferative genes (e.g., cyclin D1, c-Myc). In RA synovial tissue, this cascade drives synovial fibroblast proliferation, pannus formation, and cartilage/bone erosion. For preferential JAK1 inhibitors, targeted inhibition of JAK1 disrupts IL-6/STAT3 signaling (key for Th1/Th17 cell activation) while sparing JAK2-mediated hematopoietic signaling and JAK3-dependent lymphocyte survival, thereby inhibiting RA pathology without excessive off-target toxicities. Therefore, preferential JAK1 inhibitors hold a significant potential advantage (Blauvelt et al., 2022; Cheng et al., 2025; Peeva et al., 2018; Zhang et al., 2026; Zhu et al., 2022).

LW402 was a preferential JAK1 inhibitor. In enzymatic assays, LW402 exhibited a potent JAK1 inhibitor with an IC_50_ of 7.7 nM. The selectivity over JAK2, JAK3, and TYK2 was 1.65-, 22.85-, and 29.48-fold, respectively (Zhang et al., 2021). In collagen-induced mouse arthritis and adjuvant-induced rat arthritis models, LW402 significantly reduced multiple arthritis-related scores in a dose-dependent manner. By preferentially modulating JAK1 activity, LW402 is expected to maintain efficacy in inflammatory and autoimmune diseases while potentially reducing the hematological toxicities linked to JAK2 blockade and immunological toxicities associated with JAK3 inhibition. Pharmacokinetic studies showed that LW402 is well absorbed orally and widely distributed in Sprague-Dawley (SD) rats, with no obvious concentration-dependent or species-dependent differences in plasma protein binding. LW402 was metabolized and excreted mainly as the parent drug LW402 and its metabolite LW40241 (>10% of parent drug) in SD rats. Toxicological studies reveal that the no observed adverse effect level (NOAEL) of LW402 in a 4-week repeated-dose toxicity study in SD rats was 100 mg/kg, while the NOAEL in a 4-week repeated-dose toxicity study in cynomolgus monkeys was 30 mg/kg. These favorable preclinical studies guaranteed the approval of the clinical trial from the National Medicinal Product Agency (NMPA) of China; the present study sought to characterize the safety, tolerability, pharmacokinetic, and pharmacodynamic profiles of LW402 in healthy Chinese male participants.

## Methods

### Study design

**FIGURE 1:**
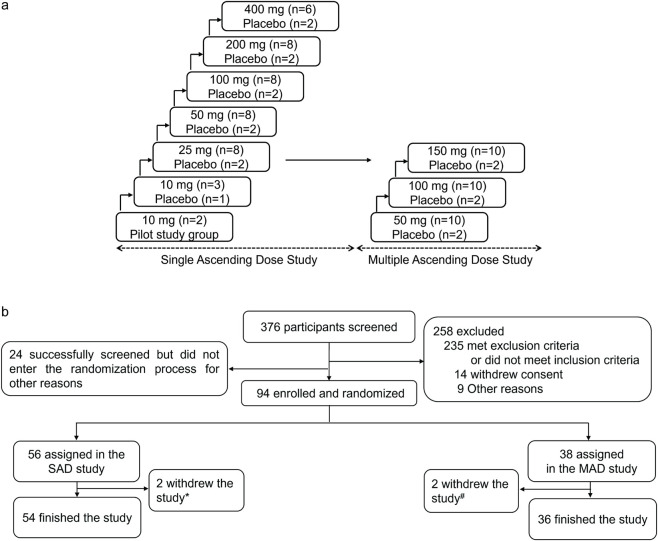
Study design and CONSORT diagram. **(a)** Study design. **(b)** CONSORT diagram. Note: *One subject in the 10 mg group withdrew from the trial before the first dose due to abnormal vital signs; One subject in the 25 mg group withdrew from the trial before the first dose due to the judgment of the physician that were not suitable for the subject to continue the study. ^#^Two study participants in the placebo group withdrew from the study due to needle phobia.

This single-center, randomized, double-blind, placebo-controlled trial was designed to assess the safety, tolerability, pharmacokinetics, and pharmacodynamics of LW402 tablets after single and multiple ascending doses in healthy Chinese male volunteers. The trial included two parts: a single ascending dose (SAD) study and a multiple ascending dose (MAD) study. The SAD portion examined six dose levels (10, 25, 50, 100, 200, and 400 mg), while the MAD portion evaluated three dose levels (50, 100, and 150 mg, b.i.d for 6 days and a single dose administration on day 7). The overall study design is illustrated in Figure 1a.

### Ethics

This trial was conducted at the Phase I Clinical Trial Center of Shanghai Xuhui Central Hospital between November 2020 and February 2022. It was registered (CTR20201897) at http://www.chinadrugtrials.org.cn. The study protocol received approval from the hospital’s Ethics Committee (No. 2020-037) and was carried out in accordance with the Declaration of Helsinki and Good Clinical Practice guidelines. Before any study procedures, all participants provided written informed consent.

### Participants

Eligible participants were healthy male volunteers aged 18–60 years. Key inclusion criteria required a body weight of ≥50.0 kg, a body mass index (BMI) between 19.0 and 25.0 kg/m^2^. Volunteers meeting any exclusion criteria were ineligible. Key exclusion criteria were described in the Supplementary Material.

### Dose level justification

LW402 tablets (10 mg, 25 mg, 100 mg) and matching placebo were supplied by Yaoyuan Biotechnology (Qidong) Co., Ltd. Based on the NMPA’s “Guidelines for Estimating the Maximum Recommended Starting Dose of Drugs in the First Clinical Trial of Healthy Adult Volunteers” (CDE, 2012), FDA’s “Guidance for Industry: Estimating the Maximum Safe Starting Dose in Initial Clinical Trials for Therapeutics in Adult Healthy Volunteers” (US-FDA, 2005), The maximum recommended starting dose (MRSD) of LW402 was calculated as 57.6 mg (based on NOAEL in cynomolgus monkeys: 30 mg/kg; human equivalent dose [HED] = 30 × 0.32 = 9.6 mg/kg; 9.6 × 60 kg/10 = 57.6 mg). To ensure safety, the starting dose was set at 10 mg (17% of MRSD). Intermediate doses (25 mg, 50 mg, 100 mg, 200 mg) were selected to evaluate dose-proportionality, with intervals based on preclinical data showing linear exposure up to 100 mg/kg in rats. The 400 mg dose was determined based on preclinical toxicology studies (NOAEL = 30 mg/kg in cynomolgus monkeys; NOAEL = 100 mg/kg in SD rats; HED = 576 mg and 972 mg, respectively). For the MAD study, 50 mg, 100 mg, and 150 mg b.i.d. were selected based on the minimum expected biological effect dose (54.2 mg b.i.d.) and median clinically effective dose (75 mg b.i.d.) predicted from preclinical PD models.

### Objectives

The primary aim of this study was to evaluate the safety and tolerability of single and multiple oral doses of LW402 tablets in healthy Chinese male volunteers. Secondarily, it sought to characterize the pharmacokinetic and pharmacodynamic profiles of LW402. Exploratory objectives included investigating possible drug metabolites and further exploring their pharmacodynamic characteristics in this population.

### Randomization and masking

To ensure double-blind conditions, participants, investigators, and all trial-related personnel remained unaware of treatment assignments throughout the study. After providing written informed consent, eligible participants received sequential screening numbers. Randomization codes were generated by an independent statistician using a block randomization scheme via the PLAN procedure in SAS software (version 9.4).

### Procedures

For the SAD study, participants were admitted 1 day before dosing. On Day 1, after a >10-h overnight fast, they received a single dose of LW402 or placebo with 240 mL of water. They remained in the center for 48 h post-dose, and a telephone follow-up was completed on Day 7. The MAD study followed a similar admission and fasting protocol. Dosing was performed once daily for 7 days, with each administration occurring within 30 min after the fasting period. Participants were confined to the center until 48 h after the final dose, and a follow-up call was made within the subsequent 7 days.

### Safety and tolerability assessment

Safety assessments included monitoring of vital signs (blood pressure, heart rate, respiratory rate, body temperature), physical examinations, clinical laboratory tests, 12-lead ECGs, and adverse events (AEs) throughout the trial. All AEs were graded (Grade 1–5) using the CTCAE v5.0, promptly managed and recorded by investigators, and coded with MedDRA v23.1. The incidence of AEs was calculated as the proportion of participants experiencing at least one AE. Treatment-emergent adverse events (TEAEs) were defined as those occurring from the first dose to the completion of follow-up. TEAEs were considered drug-related if assessed as having a causal relationship (related, probably related, or possibly related) to the investigational drug. In cases where the relationship was undetermined or missing, the event was conservatively included in the drug-related TEAE summary. In the SAD study, the termination criteria were determined by AEs, when 1) in case of serious safety issues during the study, 2) if significant flaws were found in the clinical study protocol or if there were major deviations in the implementation of the designed protocol, 3) when the sponsor requests termination 4) when the National Medical Products Administration orders the revocation of the trial for certain reasons. The termination criteria for the MAD study were consistent with those of the SAD study.

### Pharmacokinetic assessment

For pharmacokinetic assessment, blood samples were collected at specified times before and after administration. In the SAD study, 3 mL heparin-anticoagulated whole blood was collected within 30 min pre-dose (Day 1) and at multiple post-dose time points (0.25 h, 0.5 h, 1.0 h, 1.5 h, 2.0 h, 3.0 h, 4.0 h, 6.0 h, 8.0 h, 12 h, 24 h [D2], 48 h [D3]) for the 10 mg cohort; for the 25–400 mg cohorts, sampling time points included pre-dose (Day 1, within 30 min) and 0.167 h, 0.33 h, plus the same post-dose time points as the 10 mg cohort up to 48 h (D3). For the MAD study, 3 mL of heparin sodium anticoagulated whole blood samples were collected at predose (day 1 within 30 min) and at 0.167 h, 0.33 h, 0.5 h, 1 h, 1.5 h, 2 h, 3 h, 4 h, 6 h, 8 h, 12 h, 24 h (day 2 predose) and day 4, day 5 and day 6 (predose within 30 min), predose (day 7 within 30 min) and at 0.167 h, 0.33 h, 0.5 h, 1 h, 1.5 h, 2 h, 3 h, 4 h, 6 h, 8 h, 12 h, 24 h (day 8), 36 h (day 8) and 48 h (day 9). Following collection, samples were centrifuged at 4 °C and 1,500 g for 10 min. The resulting plasma was aliquoted into two tubes and stored at −80 °C until analysis. Urine samples were collected pre-dose (within 2 h) and over intervals of 0–4, 4–8, 8–12, 12–24, and 24–48 h post-dose in the single-dose study.

Given that LW402 is metabolized and excreted primarily as the parent drug LW402 and its major metabolite LW40241 (accounting for >10% of the parent drug exposure), the concentrations of LW402 and LW40241 in plasma and urine were quantified using a validated in-house LC-MS/MS method (unpublished), with LW402-d5 and LW40241-d5 as stable isotope-labeled internal standards. The linear ranges were 2–2,000 ng/mL for LW402 and 20–7,000 ng/mL for LW40241. The within-run precision and between-run precision did not exceed 9.3% and 13.7%, respectively.

Cumulative excretion (mg) and excretion rate (%) in urine were calculated by multiplying urine concentration (ng/mL) by urine volume (mL) and normalizing to the administered dose.

### Pharmacodynamic assessment

To assess pharmacodynamics, an *ex vivo* induction assay was developed to measure the inhibitory effect of LW402 on interleukin-6 (IL-6)-induced phosphorylated signal transducer and activator of transcription-3 (pSTAT3) and granulocyte-macrophage colony-stimulating factor (GM-CSF)-induced phosphorylated signal transducer and activator of transcription-5 (pSTAT5) signaling. For this purpose, blood samples were collected at specified times. In the SAD study, 4 mL of heparin-anticoagulated blood was collected at pre-dose (Day 1) and 1, 3, 6, 12, and 24 h post-dose for the 10 mg cohort; for the 25–400 mg cohorts, sampling was performed at pre-dose and 1, 3, 8, and 24 h post-dose. For the MAD study, blood samples were obtained at pre-dose on Day 1 and Day 7, with additional sampling at 1, 3, 8, and 24 h after the Day 7 dose. The whole blood samples were stimulated with the addition of IL-6 or GM-CSF. Peripheral blood mononuclear cells (PBMCs) were then isolated, lysed, and analyzed for pSTAT3 and pSTAT5 levels using a validated in-house method (unpublished). The inhibition rate was calculated as: (pre-dose level - post-dose level)/pre-dose level × 100%. The within-run precision and between-run precision of pSTAT3 and pSTAT5 were <30%, respectively.

### Statistical analysis

For the safety analysis, treatment-emergent adverse events (TEAEs) were summarized by event count, incidence rate, and number of affected participants. Descriptive statistics are presented as mean and coefficient of variation (CV%). For the pharmacokinetic (PK) analysis, primary PK parameters, including AUC_0–24h_, AUC_0–t_, ${\text{AUC}}_{0-\infty }$ , C_max_, t_max_, t_1/2_, K_el_, V_z/F_, CL/F, and MRT were derived via non-compartmental analysis (NCA) using Phoenix WinNonlin (v8.3). C_max_ and t_max_ were obtained directly from observed data. Cumulative excretion volume, excretion rate, and renal clearance were calculated separately. For the MAD study, the degree of fluctuation (DF) and accumulation ratio (AR) at steady state were also determined. Dose proportionality was evaluated using a power model: ln (PK) = α + β × ln (Dose). Dose proportionality for C_max_, AUC_0-t_, and ${\text{AUC}}_{0-\infty }$ was concluded if the 90% confidence interval for β fell within 0.8–1.25. For pharmacodynamic (PD) Analysis, observed values, changes from baseline, and inhibition rates (calculated as [pre-dose–post-dose]/pre-dose × 100%) at each time point were summarized using descriptive statistics and graphically presented. Inter-group comparisons were performed using analysis of variance (ANOVA).

## Results

### Demographic profile

A total of 94 eligible participants were enrolled and randomized into the study: 56 into the SAD part and 38 into the MAD part (Figure 1b). In the SAD cohort, two participants withdrew before dosing—one (10 mg group) due to abnormal vital signs and another (25 mg group) based on the investigator’s judgment. Consequently, 54 participants completed the SAD study. In the MAD cohort, two participants in the placebo group withdrew owing to needle phobia; the remaining 36 participants completed the study. Baseline demographic characteristics, including age, sex, ethnicity, and body mass index (BMI), were comparable across all treatment groups (Table 1).

**TABLE 1:** Baseline demographic characteristics.

​	SAD study							MAD study			
Dose, mg	10	25	50	100	200	400	P	50	100	150	P
n	6	9	8	8	8	6	11	10	10	10	8
Age, y											
Mean	28.8	28.9	26.3	30.5	26.1	30.3	26.5	26.0	26.5	24.3	29.1
SD	1.9	5.0	2.7	5.8	2.4	6.6	4.9	5.9	4.9	4.1	4.9
Sex, %											
Male	100	100	100	100	100	100	100	100	100	100	100
Female	0	0	0	0	0	0	0	0	0	0	0
Ethnicity, %											
Han	83.3	100	87.5	100	100	92.0	81.8	90	100	90	100
Others	16.7	0	12.5	0	0	7.1	18.2	10	0	10	0
BMI, kg/m^2^											
Mean	22.43	21.90	22.41	22.64	22.51	21.73	21.94	23.08	22.84	22.29	22.10
SD	1.914	1.329	1.808	1.670	1.804	1.407	1.497	1.336	1.196	1.833	1.525

SAD: single ascending dose; MAD: multiple ascending dose; P: placebo; BMI: body mass index; SD: standard deviation.

### Safety and tolerability

In the SAD study, treatment-emergent adverse events (TEAEs) were reported in all LW402 dose cohorts. The overall incidence of drug-related TEAEs generally exhibited an increasing trend with escalating dose levels. The most commonly reported drug-related adverse events were cardiac disorders, including sinus bradycardia, sinus tachycardia, first-degree atrioventricular block, and intraventricular conduction disturbance, followed by investigations such as decreased blood pressure and shortened electrocardiogram PR interval, as well as nervous system disorders, including headache, dizziness, and amaurosis, and gastrointestinal disorders including diarrhea. All drug-related TEAEs were self-limiting and resolved without intervention. A single case of grade 3 TEAE (syncope) was reported in the 400 mg dose cohort and was considered possibly related to the study drug. No TEAEs of grade 4 or higher were observed. Detailed data were presented in Table 2.

**TABLE 2:** Treatment-emergent adverse events (TEAEs) of the SAD study.

Groups	TEAEs by SOC (n)	TEAEs by PT (n)
Placebo	Cardiac disorders (1)	Sinus bradycardia (1)
	Investigations (1)	Electrocardiogram PR interval shortened (1)
	Infections and infestations (1)	Subcutaneous abscess (1)
	General disorders and administration site conditions (1)	Fatigue (1)
10 mg	Investigations (1)	Electrocardiogram PR interval shortened (1)
25 mg	Cardiac disorders (2)	First degree atrioventricular block (1), Intraventricular conduction defect (2)
	Investigations (2)	White blood cell count decreased (1), Conjugated bilirubin increased (1), Blood bilirubin increased (1), Neutrophil count decreased (1)
50 mg	Cardiac disorders (1)	Sinus bradycardia (1)
	Investigations (1)	Urine leukocytes positive (1)
	Renal and urinary disorders (1)	Haematuria (1)
100 mg	Cardiac disorders (3)	Sinus bradycardia (3)
	Investigations (1)	Blood uric acid increased (1)
	Gastrointestinal disorders (2)	Diarrhoea (2), Abdominal pain (1)
200 mg	Cardiac disorders (2)	Sinus bradycardia (2)
400 mg	Cardiac disorders (3)	Sinus tachycardia (2), First degree atrioventricular block (1), Palpitations (1)
	Investigations (4)	Blood pressure decreased (4), Blood creatine phosphokinase increased (1)
	Nervous system disorders (4)	Headache (4), Dizziness (2), Somnolence (1), Cramp-fasciculation syndrome (1), Syncope (1)
	Eye disorders (3)	Amaurosis (2), Periorbital oedema (1)
	Vascular disorders (1)	Flushing (1)

SOC: system organ class; PT: preferred term.

During the MAD phase, drug-related TEAEs were observed across all LW402 treatment groups. The most frequent drug-related adverse events were mainly investigations, including elevated serum uric acid, increased lymphocyte count, elevated liver enzymes (alanine aminotransferase, aspartate aminotransferase, and γ-glutamyl transferase), as well as gastrointestinal disorders such as oral ulcer and oropharyngeal pain. Most TEAEs were mild (grade 1) in severity, with a small proportion being moderate (grade 2). No TEAEs of grade 3 or higher were reported during the MAD phase. Detailed data were presented in Table 3.

**TABLE 3:** Treatment-emergent adverse events (TEAEs) of the MAD Study.

Groups	TEAEs by SOC (n)	TEAEs by PT (n)
Placebo	Investigations (2)	Blood creatine phosphokinase increased (1), Blood cholesterol increased (1)
	Gastrointestinal disorders (1)	Oral ulceration (1)
	Respiratory, thoracic and mediastinal disorders (1)	Oropharyngeal pain (1)
	Cardiac disorders (1)	Intraventricular conduction defect (1)
	Musculoskeletal and connective tissue disorders (1)	Back pain (1)
50 mg b.i.d	Investigations (4)	Blood uric acid increased (1), Alanine aminotransferase increased (1), Aspartate aminotransferase increased (1), Gamma-glutamyltransferase increased (2), Blood creatine phosphokinase increased (1), Total bile acid increased (1)
	Gastrointestinal disorders (1)	Oral ulceration (1)
	Respiratory, thoracic and mediastinal disorders (1)	Oropharyngeal pain (1)
	Cardiac disorders (1)	Sinus bradycardia (1)
	Infections and infestations (1)	Pharyngitis (1)
100 mg b.i.d	Investigations (4)	Lymphocyte count increased (2), Alanine aminotransferase increased (1), Aspartate aminotransferase increased (1), White blood cell count increased (1), Conjugated bilirubin increased (1), Blood bilirubin increased (1), Blood glucose decreased (1), Neutrophil count increased (1)
	Gastrointestinal disorders (4)	Oral ulceration (2), Constipation (1), Abdominal distension (1), Gingival bleeding (1)
	Respiratory, thoracic and mediastinal disorders (1)	Dry throat (1)
150 mg b.i.d	Investigations (3)	Blood uric acid increased (1), Blood triglycerides increased (1), Neutrophil count decreased (1)

SOC: system organ class; PT: preferred term; b.i.d for 6 days and a single dose administration on Day 7.

### Pharmacokinetic analysis

**FIGURE 2:**
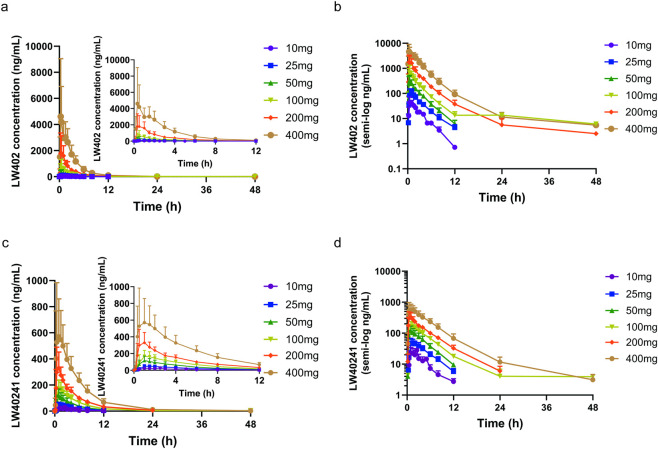
Mean plasma drug concentration-time curves of the SAD study. **(a)** LW402 of 0–48 h and 0–12 h segment. **(b)** LW402 of 0–48 h in semi-log. **(c)** 0- LW40241 of 0–48 h and 0–12 h segment. **(d)** LW40241 of 0–48 h in semi-log.

The mean plasma concentration-time profiles and the primary pharmacokinetic parameters of LW402 following single-dose administration are presented in Figure 2 and Table 4, respectively. The median of time to maximum plasma concentration (t_max_) was 0.42–1.0 h in the dose cohorts. The mean of the C_max_ and the AUC_0–t_ were 49.44–6056.83 ng/mL and 135.26–13594.13 h*ng/mL, respectively. In the dose range of 10–400 mg, the increases in C_max_, AUC_0-t_, and ${\text{AUC}}_{0-\infty }$ of LW402 were slightly more than proportional to the dose (Supplementary Figures 1a,b). This was determined via power model analysis (ln (PK) = α + β × ln (Dose)), where the slope β for C_max_ was 1.18 (90% CI: 1.05–1.32) and for AUC_0–t_ was 1.21 (90% CI: 1.08–1.35), exceeding the upper limit of 1.25 for strict dose-proportionality. However, the deviations were mild (<25%), indicating near-dose-proportional exposure. Although the slope β slightly exceeded 1.25, the 90% confidence interval for the slope was within the accepted range for near dose proportionality as defined by regulatory guidelines. The observed deviation was minor and not clinically meaningful. Subject S4003 was excluded as an outlier based on predefined statistical criteria (e.g., Grubbs’ test) due to exceptionally high exposure that was inconsistent with the overall PK profile and considered non-representative of the study population.

**TABLE 4:** Pharmacokinetic parameters of LW402 in the single-ascending-dose study.

Parameters (Units)	10 mg (n = 5)	25 mg (n = 8)	50 mg (n = 8)	100 mg (n = 8)	100 mg (n = 7)^b^	200 mg (n = 8)	400 mg (n = 6)
${\text{AUC}}_{0-\infty }$ (h*ng/mL)	144.41 (25.4)	377.81 (20.8)	871.97 (22.3)	1807.52 (19.9)	1874.58 (17.6)	4970.20 (18.2)	13654.39 (30.7)
AUC_0-t_ (h*ng/mL)	135.26 (23.6)	360.75 (21.6)	850.71 (22.3)	1753.70 (21.7)	1835.87 (17.7)	4946.01 (18.2)	13594.13 (31.1)
CL/F (L/h)	72.46 (22.2)	69.00 (22.8)	59.86 (21.7)	57.79 (24.7)	55.37 (24.4)	41.48 (19.0)	32.14 (35.7)
C_max_ (ng/mL)	49.44 (47.2)	129.47 (24.9)	369.15 (41.4)	848.86 (62.2)	952.87 (49.7)	2557.25 (39.3)	6056.83 (48.3)
${\text{MRT}}_{0-\infty }$ (h)	3.19 (8.4)	3.64 (14.6)	3.18 (14.0)	5.13 (115.3)	3.04 (16.8)	3.61 (29.5)	3.69 (23.1)
MRT_0-t_ (h)	2.68 (10.3)	3.12 (12.2)	2.88 (14.2)	3.98 (85.6)	2.79 (16.7)	3.46 (27.7)	3.51 (21.7)
t_1/2_ (h)	2.01 (13.5)	2.45 (18.6)	2.29 (10.8)	4.21 (134.0)	2.22 (6.4)	4.09 (43.2)	4.19 (48.8)
t_max_^a^ (h)	1 (0.5, 1.5)	0.75 (0.5, 2)	0.5 (0.5, 2)	0.75 (0.333, 2)	0.5 (0.333, 1.5)	0.75 (0.333, 2)	0.42 (0.333, 1.5)
V_z_/F (L)	208.89 (25.6)	237.29 (14.8)	197.14 (23.6)	399.86 (158.0)	176.87 (23.7)	249.14 (54.2)	221.79 (88.4)
λ_z_ (1/h)	0.35 (15.2)	0.29 (21.1)	0.31 (11.5)	0.28 (35.5)	0.31 (6.5)	0.19 (25.9)	0.19 (35.0)

Data are expressed as mean (%CV), except for t_max_^a^, which is shown as median (min, max).

^b^ This is the sensitivity analysis result of the 100 mg dose group after excluding S4003.

The pharmacokinetic parameters (MRT, t_1/2_, Vz/F, CL/F) of the metabolite LW40241 were consistent across the eight dose groups (Table 5). Following a single dose, LW40241 was rapidly absorbed, with a median t_max_ ranging from 0.76 to 1.25 h, a profile similar to that of the parent drug, LW402. Both the mean C_max_ and AUC_0-t_ increased with the dose range of 10–400 mg. Strong linear correlations with dose were observed, as evidenced by correlation coefficients (R^2^) of 0.8990 for C_max_ and 0.9562 for AUC_0–t_ (Supplementary Figures 1c,d). These results indicated that within the dose range of 10–400 mg, AUC_0–t_ of LW40241, and the dose range of 50–200 mg, C_max_ of LW40241 increase in proportion to the dose. The increasing proportions of the remaining exposure parameters are slightly lower than that of the dose.

**TABLE 5:** Pharmacokinetic parameters of LW40241 in the single-ascending-dose study.

Parameters (Units)	10 mg (n = 5)	25 mg (n = 8)	50 mg (n = 8)	100 mg (n = 8)	100 mg (n = 7)^b^	200 mg (n = 8)	400 mg (n = 6)
${\text{AUC}}_{0-\infty }$ (h*ng/mL)	117.30 (38.9)	295.26 (17.7)	583.53 (21.1)	1045.09 (15.1)	1057.92 (15.7)	1746.42 (15.5)	3719.03 (26.2)
AUC_0-t_ (h*ng/mL)	103.55 (41.3)	266.36 (18.0)	542.29 (22.3)	1014.75 (17.3)	1035.74 (17.3)	1706.17 (15.9)	3660.98 (26.3)
AUC_%Extrap_ (%)	12.38 (34.9)	9.85 (26.7)	7.35 (29.9)	3.21 (99.7)	2.36 (96.8)	2.33 (55.5)	1.59 (69.4)
C_max_ (ng/mL)	23.65 (44.8)	57.02 (26.7)	127.43 (27.4)	185.08 (33.6)	200.83 (23.3)	382.29 (33.7)	688.55 (44.2)
${\text{MRT}}_{0-\infty }$ (h)	5.13 (13.4)	5.60 (11.4)	4.91 (9.4)	7.06 (62.1)	5.52 (10.3)	5.97 (16.3)	6.41 (23.9)
MRT_0-t_ (h)	3.77 (11.5)	4.35 (7.4)	3.99 (7.6)	6.01 (45.1)	5.07 (13.0)	5.39 (12.6)	5.93 (22.6)
T_1/2_ (h)	3.25 (29.0)	3.43 (13.1)	3.01 (13.3)	5.16 (80.7)	3.69 (9.4)	4.43 (14.2)	4.78 (30.3)
t_max_^a^ (h)	1 (1, 2)	1.25 (1, 3)	1 (1, 3)	1 (0.5, 3)	1 (0.5, 3)	1 (0.5, 2)	0.76 (0.5, 2)

Data are expressed as mean (%CV), except for t_max_^a^, which is shown as median (min, max).

^b^ This is the sensitivity analysis result of the 100 mg dose group after excluding S4003.

**FIGURE 3:**
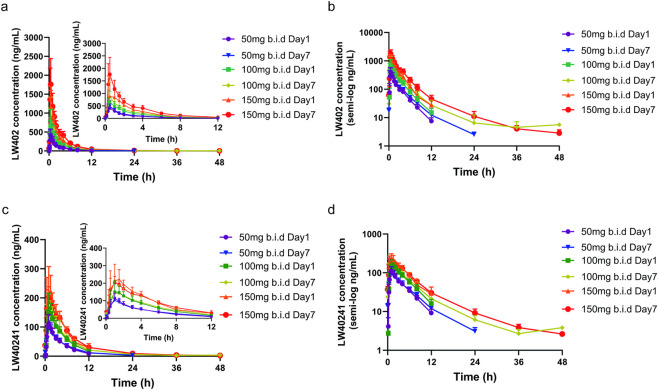
Mean plasma drug concentration-time curves of the MAD study. **(a)** LW402 of 0–48 h and 0–12 h segment. **(b)** LW402 of 0–48 h in semi-log. **(c)** 0- LW40241 of 0–48 h and 0–12 h segment. **(d)** LW40241 of 0–48 h in semi-log.

The mean steady-state plasma concentration-time profiles and pharmacokinetic parameters for the MAD study are presented in Figure 3 and Tables 6, 7. For LW402, steady-state exposure measures (C_max_, C_ss_min_, and AUC_0-t_) increased with dose. Accumulation was observed after multiple doses, with accumulation ratios ranging from 1.23 to 1.79 for C_max_ and 1.25 to 1.52 for AUC_0–t_. The mean elimination half-life (t_1/2_) ranged from 3.98 to 5.70 h. Compared with single-dose administration, parameters governing elimination (Vz/F, CL/F, t_1/2_) showed no significant change, indicating consistent elimination characteristics upon repeated dosing. Dose-linearity assessment using confidence interval criteria revealed near dose-proportional increases, with slopes of 1.206 for C_ss, max_ vs. dose and 1.187 for AUC_0–48h_ vs. dose on Day 7 (Supplementary Figures 2a,b); the slope of C_ss, max_ vs. dose and AUC_0–48h_ of LW40241 vs. dose was 0.6238 and 0.7578 (Supplementary Figures 2c,d). The correlation coefficient R^2^ of C_ss, max,_ and AUC_0–48h_ was 0.609 and 0.8318 for LW402 (Day7), and 0.505 and 0.7233 for LW40241 (Day7), respectively. Therefore, the C_ss, max,_ and AUC_0–48h_ were approximately dose-dependent in the dose range of 50–150 mg b.i.d in the MAD study. AUC_(0–48h)_ was used for the MAD study since only a single dose administration on day 7 (excluding the routine b.i.d. second dose), which fully captures the complete systemic exposure of LW402 and its metabolite LW40241 after the final dose, consistent with the study’s actual sampling design and facilitating accurate evaluation of the steady-state of pharmacokinetic parameters.

**TABLE 6:** Pharmacokinetic parameters of LW402 in the multiple-ascending-dose study.

LW402			50 mg b.i.d (n = 10)	100 mg b.i.d (n = 10)	150 mg b.i.d (n = 10)
Day	Parameters	Units	Mean (%CV)		
1	AUC_0-t_	h*ng/mL	896.21 (14.4)	1782.74 (25.1)	2764.33 (14.3)
	C_max_	ng/mL	443.64 (24.3)	941.16 (48.9)	1190.30 (41.3)
	t_1/2_	h	2.38 (8.2)	2.34 (12.7)	2.12 (8.2)
	t_max_^a^	h	0.5 (0.333, 1.5)	0.5 (0.333, 1.5)	0.75 (0.333, 2)
	λ_z_	1/h	0.29 (8.3)	0.30 (10.4)	0.33 (8.7)
7	AR	<none>	1.14 (4.2)	1.21 (15.9)	1.31 (14.0)
	AR (AUC)	<none>	1.25 (16.3)	1.31 (17.0)	1.52 (13.0)
	AR (C_max_)	<none>	1.23 (40.8)	1.22 (28.6)	1.79 (30.8)
	AUC_0-τ_	h*ng/mL	1114.59 (18.5)	2357.40 (30.0)	4183.11 (16.1)
	AUC_0- t_	h*ng/mL	1196.57 (19.6)	2567.94 (26.6)	4600.91 (17.2)
	C_ss_avg_	ng/mL	92.88 (18.5)	196.45 (30.0)	348.59 (16.1)
	CL_ss_/F	L/h	46.04 (15.6)	47.81 (46.1)	36.65 (15.0)
	C_ss_max_	ng/mL	511.80 (31.4)	1147.87 (52.6)	1960.40 (25.2)
	C_ss_min_	ng/mL	12.31 (31.7)	23.63 (36.9)	43.52 (37.5)
	DF	%	530.94 (18.5)	540.46 (34.7)	556.17 (27.2)
	t_1/2_	h	3.98 (15.2)	4.55 (43.3)	5.70 (33.3)
	t_ss_max_^a^	h	0.5 (0.333, 1.5)	0.5 (0.333, 1.5)	0.5 (0.167, 1.5)
	V_ss_/F	L	264.65 (23.3)	357.78 (105.5)	301.41 (36.8)
	λ_z_	1/h	0.18 (18.5)	0.17 (36.9)	0.14 (39.8)

Data are expressed as mean (%CV), except for t_max_^a^ and t_ss_max_^a^, which is shown as median (min, max). B.i.d for 6 days and a single dose administration on Day 7.

**TABLE 7:** Pharmacokinetic parameters of LW40241 in the multiple-ascending-dose study.

LW40241			50 mg b.i.d (n = 10)	100 mg b.i.d (n = 10)	150 mg b.i.d (n = 10)
Day	Parameters	Units	Mean (%CV)		
1	AUC_0-t_	h*ng/mL	480.20 (14.8)	787.47 (25.1)	1141.20 (11.8)
	C_max_	ng/mL	116.27 (21.1)	174.85 (28.9)	248.21 (23.2)
	t_1/2_	h	3.11 (9.1)	3.13 (11.7)	3.12 (8.6)
	t_max_^a^	h	1 (0.5, 2)	1 (0.5, 2)	1.5 (0.5, 3)
	λ_z_	1/h	0.22 (8.8)	0.22 (11.4)	0.22 (8.7)
7	AR	<none>	1.21 (3.8)	1.37 (17.1)	1.50 (19.3)
	AR (AUC)	<none>	1.05 (9.6)	1.05 (17.4)	1.03 (11.7)
	AR (C_max_)	<none>	0.96 (24.7)	0.97 (28.4)	0.90 (20.6)
	AUC_0-τ_	h*ng/mL	502.97 (14.9)	828.72 (28.8)	1161.23 (5.7)
	AUC_0-t_	h*ng/mL	587.07 (17.6)	1013.77 (27.0)	1484.36 (8.2)
	C_ss_avg_	ng/mL	41.91 (14.9)	69.06 (28.8)	96.77 (5.7)
	C_ss_max_	ng/mL	108.78 (18)	167.08 (33.6)	216.41 (11.5)
	C_ss_min_	ng/mL	11.90 (25.0)	19.17 (28.2)	30.02 (14.4)
	DF	%	232.51 (18.7)	208.97 (18.0)	192.49 (12.4)
	t_1/2_	h	4.71 (10.7)	6.29 (35.3)	7.49 (35.2)
	t_ss_max_^a^	h	1 (0.5, 2)	1 (0.5, 2)	1 (0.5, 2)
	λ_z_	1/h	0.15 (12.3)	0.12 (30.8)	0.10 (32.6)

Data are expressed as mean (%CV), except for t_max_^a^ and t_ss_max_^a^, which is shown as median (min, max). B.i.d for 6 days and a single dose administration on Day 7.

After a single dose of 10 mg, 25 mg, 50 mg, 100 mg, 200 mg, and 400 mg of LW402 tablets, the average cumulative excretion rates of the prototype drug LW402 in urine were 21.7%, 16.8%, 19.7%, 19.6%, 26.5%, and 26.1%, respectively. The average cumulative excretion rates of the sum of the prototype drug LW402 and the metabolite LW40241 were 55.9%, 52.7%, 57.8%, 52.7%, 58.4%, and 60.5%, respectively. Therefore, the drugs excreted from the body through urine in the form of the prototype drug LW402 and the metabolite LW40241 account for approximately half of the total administered dose.

### Pharmacodynamic analysis

**FIGURE 4:**
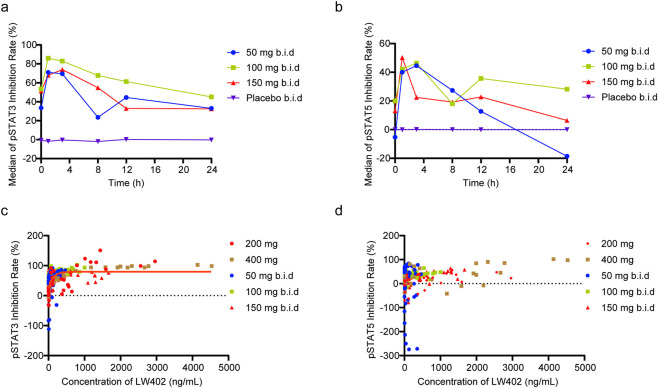
Pharmacodynamic profiles of LW402. **(a)** Median of pSTAT3 inhibition rate. **(b)** Median of pSTAT5 inhibition rate. **(c)** Correlation and data fitting model between the inhibition rates of pSTAT3 and the plasma concentration of LW402. **(d)** Correlation between the inhibition rates of pSTAT5 and the plasma concentration of LW402.

Following a single dose of LW402 (10–400 mg) or placebo, maximum inhibition of both pSTAT3 and pSTAT5 was observed at 1 h post-dose across all active dose groups, while the placebo group showed minimal to no inhibitory effect. The maximum inhibitory effect of LW402 on pSTAT3 increased in a dose-dependent manner and approached saturation at approximately 100 mg, whereas no clear dose–response relationship was observed for pSTAT5 (Table 8). The PD assessments in the SAD phase were designed as exploratory analyses for LW402. The multiple ascending dose (MAD) phase PD data, which reflect the pharmacodynamic profile under steady-state exposure, are considered more reliable and informative for characterizing LW402’s preferential JAK1 inhibitory activity. After multiple doses of LW402 (50, 100, and 150 mg b.i.d for 6 days and a single dose administration on day 7), the maximum median inhibition rates for pSTAT3 were higher than those of pSTAT5, showing the preferential inhibitory effect over pSTAT3 (Table 8; Figures 4a,b).

**TABLE 8:** Maximum median inhibition rates of pSTAT3 and pSTAT5 after LW402 administration.

Study part	SAD study						MAD study		
Dose, mg	10	25	50	100	200	400	50	100	150
pSTAT3, %	37.19	44.48	40.22	157.1	111.3	98.31	71.00	85.99	73.99
pSTAT5, %	60.33	17.80	41.03	40.70	31.11	87.91	44.57	46.27	50.25

The relationship between LW402 plasma concentration and pSTAT3 inhibition rate was well fitted with a sigmoid E_max_ model, yielding the population typical values (coefficient of variation, CV%) of key parameters as follows: E_max_ = 129.97% (25.48%), EC_50_ = 630.08 ng/mL, and γ = 0.226 (24.75%) (Figure 4c). By contrast, model fitting for the correlation between LW402 plasma concentration and pSTAT5 inhibition rate was unsuccessful, indicating a non-significant dose-effect relationship between LW402 exposure and pSTAT5 inhibition (Figure 4d). The modest and variable PD responses (pSTAT3/pSTAT5) reflect the biological heterogeneity of patient samples and the dynamic nature of signal transduction in clinical specimens. The Emax value exceeding 100% for pStat3 is a mathematical artifact derived from nonlinear regression modeling, not a true biological effect exceeding maximum inhibition. This phenomenon occasionally occurs during curve fitting when baseline variability is present and does not alter the interpretation of the drug’s PD activity.

## Discussion and conclusion

This study evaluated the safety, tolerability, pharmacokinetics, and pharmacodynamics of the preferential JAK1 inhibitor LW402 following single and multiple ascending doses in healthy volunteers. LW402 was safe and well-tolerated, with systemic exposure increasing dose-proportionally. The PD profile aligned with its mechanism of action: IL-6-induced pSTAT3 was inhibited in a dose-dependent manner, whereas GM-CSF-induced pSTAT5 was less potently inhibited compared to IL-6-induced pSTAT3. LW402 is being advanced to the Phase II clinical trials for rheumatoid arthritis (RA) and atopic dermatitis (AD), as JAK1 plays a critical role in RA and AD pathogenesis by mediating signaling of pro-inflammatory cytokines (IL-4, IL-13, IL-31).

Cardiac safety is an important class effect of JAK inhibitors. Approved JAK inhibitors, including non-selective agents, have been associated with risks of major adverse cardiovascular events such as ischemic heart disease, stroke, and venous thromboembolism, especially in patients with underlying cardiovascular risk factors (Shawky, Almalki, Abdalla, Abdelazeem and Gouda, 2022; Simpson et al., 2021). In the preclinical studies, LW402 demonstrated no significant inhibitory effect on the hERG channel, with an IC_50_ value greater than the highest tested concentration of 95.27 μM. In this first-in-human study of LW402, the most frequent cardiac-related treatment-emergent adverse events were mild and transient electrocardiographic changes, including sinus bradycardia, first-degree atrioventricular block, intraventricular conduction disturbance, and sinus tachycardia. All these events were reversible without specific treatment. No severe cardiovascular events, such as myocardial infarction, stroke, life-threatening arrhythmia, or thromboembolic events, were observed. These cardiac findings were considered mechanism-based, on-target pharmacodynamic effects rather than overt cardiac toxicity. Compared with other JAK inhibitors, LW402 exhibited a favorable and manageable cardiac safety profile in healthy participants, supporting its further clinical development (Chen et al., 2023; Huang et al., 2022; Peeva et al., 2018).

The pharmacokinetic profile of LW402 in healthy volunteers was characterized by rapid absorption and elimination. A short elimination half-life (mean range: 2–5 h) was observed across doses. This profile corresponded to a high apparent clearance (CL/F: 30–75 L/h) coupled with a large volume of distribution (Vz/F: approximately 176–400 L/h), indicative of the compound’s extensive distribution and efficient elimination from the body. In the 100 mg dose cohort, subject S4003 presented a notably smaller terminal elimination slope in the concentration-time curve (Table 4), which led to a significantly lower λz and an abnormally longer t_1/2_ compared with the other 7 participants. Further comparison indicated that the t_1/2_ of S4003 was obviously higher than that of other dose cohorts (10–50 mg), whereas the t_1/2_ of the remaining 7 participants in the 100 mg cohort was consistent with other dose groups. Since the λz-associated PK parameters of S4003 had a large deviation from the cohort and would markedly influence the statistical mean values, a sensitivity analysis of the PK parameters for the 100 mg cohort was conducted by excluding the data of subject S4003, and the results are presented in Table 4.

Steady state was achieved on Day 7 of repeated dosing for LW402 and its metabolite LW40241, since the dosing duration (7 days) was far longer than 4–5 elimination half-lives of LW402 (3.98–5.70 h at multiple doses); the trough plasma concentrations (C_ss_min_) reached a plateau without further increase with additional doses; and the accumulation ratios (AR) of C_max_ and AUC_0-t_ at steady state were low and stable (1.23–1.79 for C_max_ and 1.25–1.52 for AUC_0–t_), indicating a dynamic balance between drug absorption and elimination was established. Plasma concentrations exhibited pronounced fluctuations over the dosing interval, characteristic of drugs with short half-lives administered intermittently. LW402 also showed dose-proportional exposure (C_max_ and AUC_0-t_) with moderate individual differences, suggesting predictable exposure. The pharmacokinetic profile of LW402 was similar to that of other JAK inhibitors (Banfield et al., 2018; Qu et al., 2024; Shi et al., 2014).

As for pharmacological activity, LW40241, the major metabolite of LW402, showed potent JAK1 inhibitory activity (IC_50_ = 3.61 nM), indicating that LW40241 retains the JAK1 inhibitory profile of its parent drug. In terms of safety assessment, LW40241 was not evaluated as a standalone agent; instead, its safety was assessed in the context of parent LW402 administration in non-clinical toxicology studies. In these repeated-dose toxicity studies, systemic exposure to both LW402 and LW40241 was quantitatively measured, and the metabolite exposure levels in animals fully covered the human systemic exposure observed in this phase 1 trial. No unique or additional safety risks attributable to LW40241 were identified throughout the toxicology evaluations. Therefore, the metabolite does not introduce additional clinical safety concerns.

In this study, the JAK1-preferential inhibitor LW402 exhibited rapid and potent inhibition of IL-6-induced pSTAT3 but less potent inhibition of GM-CSF-induced pSTAT5, with maximum median inhibition rates of 157.1% (pSTAT3) and 87.9% (pSTAT5) in the SAD study, and 85.99% (pSTAT3) and 50.25% (pSTAT5) in the MAD study. Compared to tofacitinib (potency ratio for pSTAT3/pSTAT5: 0.67), LW402 showed a higher potency ratio (∼2.0), indicating approximately 2-fold greater selectivity for JAK1- over JAK2-mediated signaling and a superior safety profile potential (Banfield, Scaramozza, Zhang, Kieras, Page, Fensome, Vincent, Dowty, Goteti, Winkle and Peeva, 2018; Gong et al., 2020; Li et al., 2022; Meng et al., 2022).

Given the safety concerns associated with non-selective JAK inhibitors, particularly hematological toxicities linked to JAK2 inhibition, the rationale for investigating JAK1-preferential inhibitors like LW402 was further supported by the role of JAK2 in hematopoietic signaling. JAK2 was a critical signaling component for hematopoietic cytokines such as erythropoietin (EPO), thrombopoietin (TPO), and granulocyte colony-stimulating factor (G-CSF) (He et al., 2024). Inhibition of JAK2 disrupts the JAK-STAT cascade required for hematopoietic progenitor cell proliferation and differentiation, leading to anemia, thrombocytopenia, and neutropenia (Harrison et al., 2020; Komrokji et al., 2015). By preferentially targeting JAK1, LW402 is expected to minimize these hematological toxicities while maintaining efficacy against RA and AD, addressing an unmet need in JAK inhibitor therapy (Hoisnard et al., 2022).

This failure of model fitting for the correlation between LW402 plasma concentration and pSTAT5 inhibition rate was attributed to two factors: (1) lower inhibitory potency of LW402 on pSTAT5 (maximum median inhibition rate: 60.3% vs. 157.1% for pSTAT3), leading to insufficient dynamic range in the data; and (2) higher inter-individual variability in pSTAT5 baseline levels (CV% = 35.2% vs. 18.7% for pSTAT3), increasing data scatter. This lack of model fit did not significantly impact the study conclusions, as the primary pharmacodynamic endpoint (pSTAT3 inhibition) was well characterized and supported LW402 as a JAK1-preferential inhibitor.

The limitations of this study are as follows. First, this clinical study enrolled only male participants, with no female participants included. As this is a first-in-human (FIH) study, we adopted this design to protect healthy female participants from potential unknown safety risks. In addition, reproductive toxicity studies had not yet been completed at the time this trial was initiated, so the potential effects of the investigational product on fertility and reproduction could not be fully assessed. Therefore, only male participants were enrolled in the present study. Of note, subsequent Phase 2 clinical trials in patients with rheumatoid arthritis and atopic dermatitis will include female participants to further evaluate the safety and preliminary efficacy of LW402 in this population. Second, the sample size was relatively small, with only 56 and 38 volunteers enrolled in the SAD and MAD phases, respectively. Although the sample size was sufficient to characterize the primary safety, PK, and PD profiles of LW402 in healthy subjects as a phase 1 trial, the limited number of participants may restrict the reliability of the findings, and larger sample sizes will be required in subsequent clinical studies to confirm these results in patient populations.

The favorable safety, tolerability, pharmacokinetic and pharmacodynamic profile of LW402 observed in this first-in-human study justify advancing this JAK1 inhibitor into further clinical trials for the treatment of rheumatoid arthritis and atopic dermatitis.

## Data Availability

The raw data supporting the conclusions of this article will be made available by the authors, without undue reservation.
