# Supplementary material for: Safety, tolerability, pharmacokinetics, and pharmacodynamics of LW402, a preferential Janus kinase 1 inhibitor in healthy volunteers: a randomized, double-blinded, placebo-controlled phase 1 trial
Source: Front Pharmacol. 2026 Apr 29;17:1784350. doi: 10.3389/fphar.2026.1784350 (PMC13167479; doi:10.3389/fphar.2026.1784350)
Supplement: Supplementary file 1 [file DataSheet1.docx]

Safety, tolerability, pharmacokinetics, and pharmacodynamics of a selective Janus Kinase 1 inhibitor, LW402, in healthy volunteers: a randomized, double-blinded, placebo-controlled phase 1 trial

**------------- Supplementary information**

**Supplemental methods**

**Key exclusion criteria**

Abnormality in physical examination, laboratory tests, chest CT, and B ultrasound with clinical significance. Study participants who have severe or uncontrolled systemic diseases with evidence, as judged by the researchers (such as severe liver damage, interstitial lung disease); instability or decompensation of cardiopulmonary function; uncontrolled hypertension; or a history of chronic gastrointestinal dysfunction due to conditions such as irritable bowel syndrome, Crohn’s disease, or ulcerative colitis. Those with a history of tumors. Those with a history of rheumatic and immune diseases. Those who had any infection judged by the researcher to be clinically significant within 3 months before enrollment. Those who had been to tuberculosis-endemic areas within 2 months before enrollment. Those with a history or evidence of tuberculosis (including a positive chest CT); an occurrence of herpes simplex, herpes zoster, or chickenpox within the 3 months prior to enrollment; or a known/suspected allergy to the study drug or its components (or a general allergic disposition). Those who had any acute disease judged by the researcher to be clinically significant within 1 month before enrollment. Those with a QTcF > 450 ms in the electrocardiogram (ECG) examination during screening (Fridericia’s formula: QTcF = QT/(RR) or those with other ECG abnormalities judged by the researcher to be clinically significant. Positive serology for hepatitis B, hepatitis C, HIV, or syphilis; concomitant use of any medication within the two weeks prior to dosing; or previous participation in a clinical trial within the preceding three months. Those who had received treatment with JAK inhibitors (such as tofacitinib) or any other drugs with a similar structure before enrollment. Those who were drug or alcohol addicts, or heavy smokers.

**Supplemental Figures**


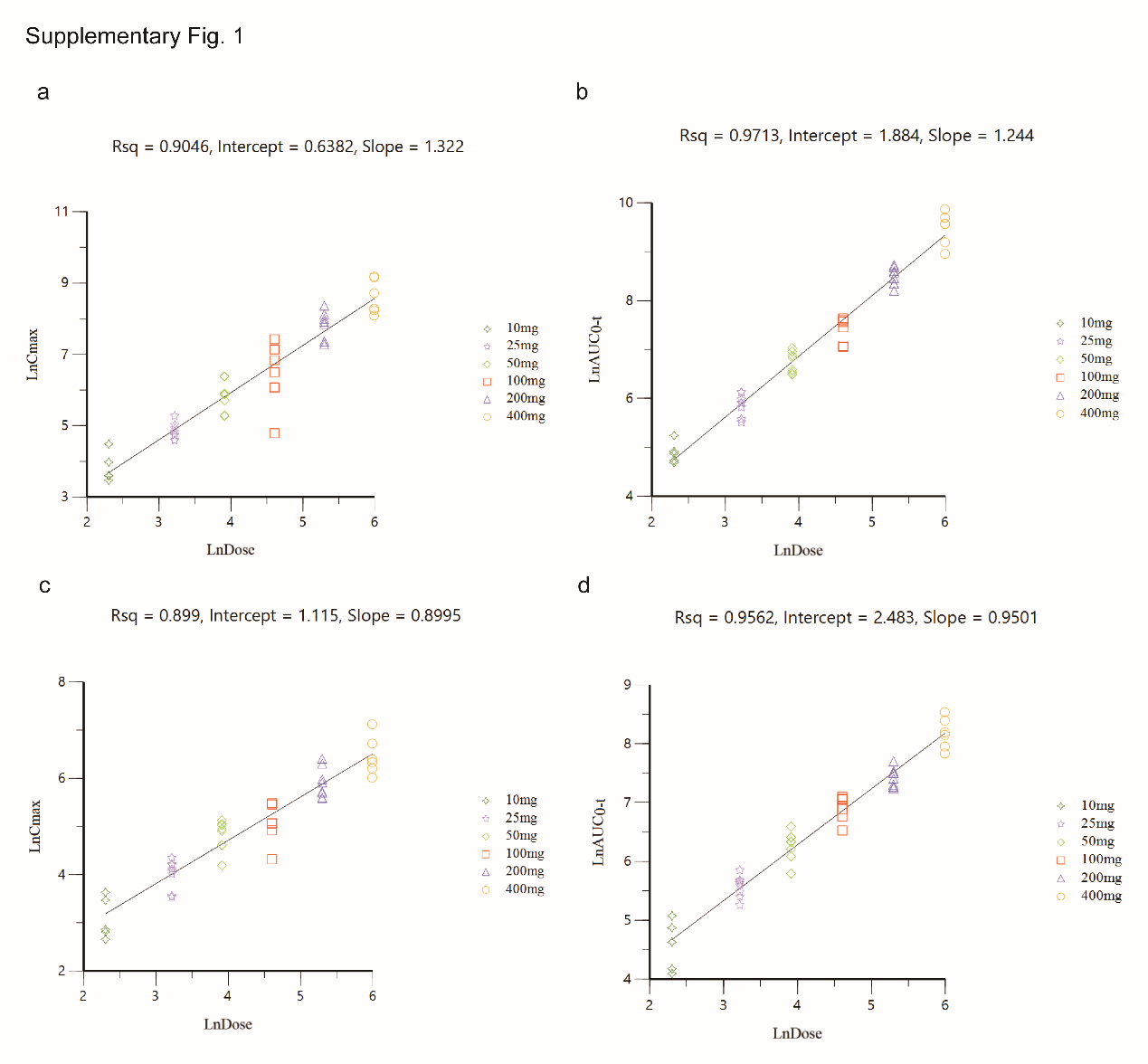


**Supplementary Figure 1.** Proportional analysis of pharmacokinetic parameters of plasma LW402 in the SAD study. **(a)** C_max_ of LW402 vs dose; **(b)** AUC_0-t_ of LW402 vs dose; **(c)** C_max_ of LW40241 vs dose; **(d)** AUC_0-t_ of LW40241 vs dose


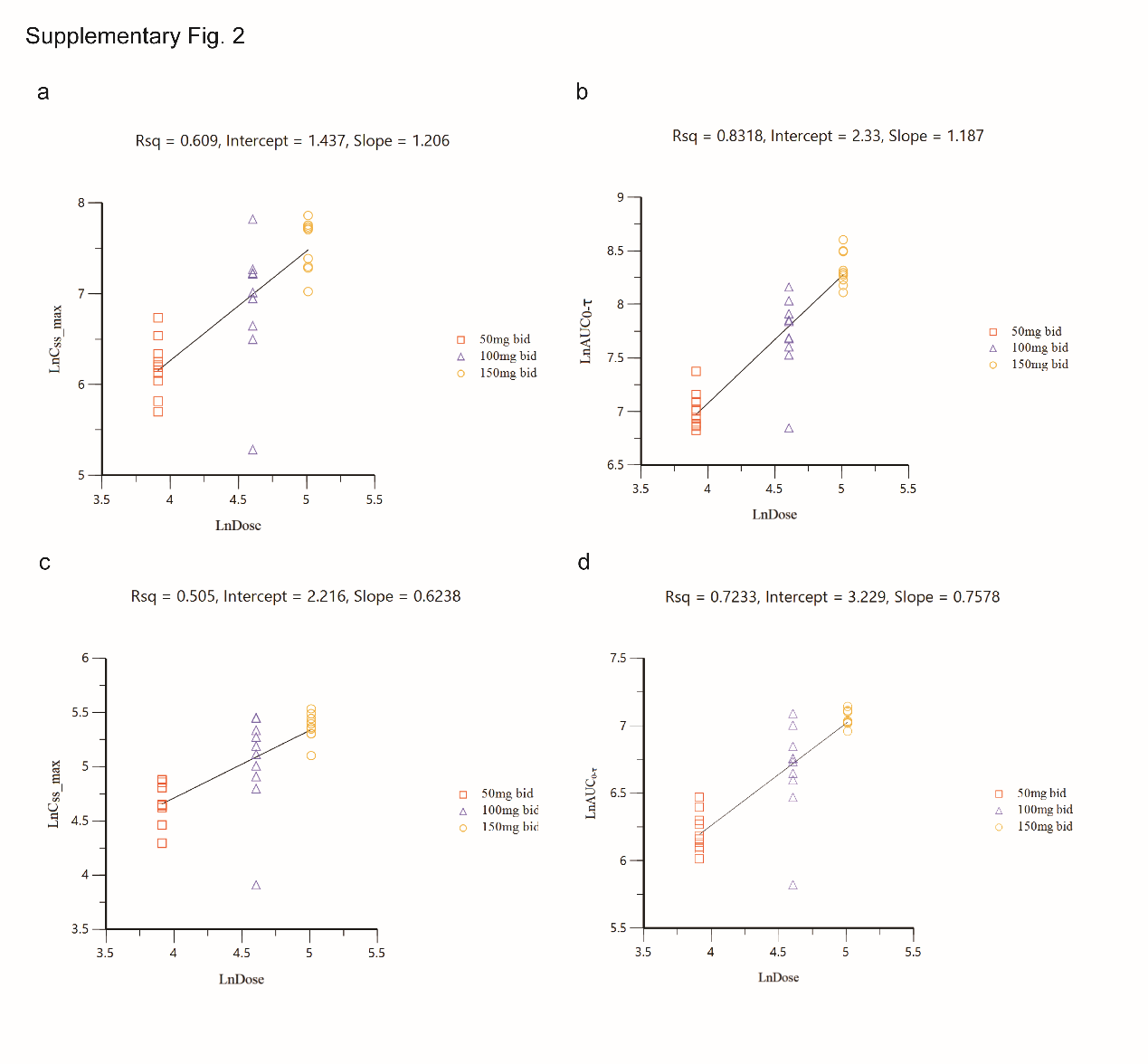


**Supplementary Figure 2.** Proportional analysis of pharmacokinetic parameters of plasma LW402 in the MAD study. **(a)** C_ss_,_max_ of LW402 vs dose; **(b)** AUC_0-t_ of LW402 vs dose; **(c)** C_ss,max_ of LW40241 vs dose; **(d)** AUC_0-t_ of LW40241 vs dose
